# HLA‐E: exploiting pathogen‐host interactions for vaccine development

**DOI:** 10.1111/cei.13292

**Published:** 2019-04-09

**Authors:** H. R. Sharpe, G. Bowyer, S. Brackenridge, T. Lambe

**Affiliations:** ^1^ Nuffield Department of Medicine Jenner Institute, University of Oxford Oxford UK; ^2^ Nuffield Department of Medicine NDM Research Building, University of Oxford Oxford UK

**Keywords:** cytomegalovirus, HLA‐E, trained immunity, vaccines

## Abstract

Viruses, when used as vectors for vaccine antigen delivery, can induce strong cellular and humoral responses against target epitopes. Recent work by Hansen *et al.* describes the use of a cytomegalovirus‐vectored vaccine, which is able to generate a stable effector‐memory T cell population at the sites of vaccination in rhesus macaques. This vaccine, targeted towards multiple epitopes in simian immunodeficiency virus (SIV), did not induce classical CD8^+^ T cells. However, non‐canonical CD8^+^ T cell induction occurred via major histocompatibility complex (MHC) class II and MHC‐E. The MHC‐E‐restricted T cells could recognize broad epitopes across the SIV peptides, and conferred protection against viral challenge to 55% of vaccinated macaques. The human homologue, human leucocyte antigen (HLA)‐E, is now being targeted as a new avenue for vaccine development. In humans, HLA‐E is an unusually oligomorphic class Ib MHC molecule, in comparison to highly polymorphic MHC class Ia. Whereas MHC class Ia presents peptides derived from pathogens to T cells, HLA‐E classically binds defined leader peptides from class Ia MHC peptides and down‐regulates NK cell cytolytic activity when presented on the cell surface. HLA‐E can also restrict non‐canonical CD8^+^ T cells during natural infection with various pathogens, although the extent to which they are involved in pathogen control is mostly unknown. In this review, an overview is provided of HLA‐E and its ability to interact with NK cells and non‐canonical T cells. Also discussed are the unforeseen beneficial effects of vaccination, including trained immunity of NK cells from bacille Calmette–Guérin (BCG) vaccination, and the broad restriction of non‐canonical CD8^+^ T cells by cytomegalovirus (CMV)‐vectored vaccines in pre‐clinical trials.

## Introduction to human leucocyte antigen‐E (HLA‐E)

### Sequence, structure and function

HLA‐E is a non‐classical, class Ib major histocompatibility complex (MHC) molecule located on chromosome 6, and is expressed throughout the majority of nucleated tissues in humans 1. Evolutionarily older than the HLA class Ia molecules (HLA‐A, ‐B and ‐C), HLA‐E distorts the traditional boundaries between fast‐acting innate and memory‐driven adaptive immunity 2. In contrast to the highly polymorphic HLA class Ia 3, HLA‐E is relatively more conserved, reducing the capacity for highly varied antigen recognition and peptide binding 4. This reduced polymorphism is also thought to confer tight constraints on binding of specific, hydrophobic epitopes 5. This alone contravenes the dogma that the high polymorphism of the classical major histocompatibility complex facilitates the recognition of (theoretically) 10^15^ epitopes, which forms the basis of adaptive immunity in vertebrates 6.

Twenty‐seven HLA‐E alleles have been reported to date, but most are found infrequently or as non‐functional proteins 7, 8, 9. HLA‐E exists predominantly in the human population as two alleles, HLA‐E*0101 and HLA‐E*0103, varying by an arginine or a glycine at amino acid position 107, respectively 10, 11. These alleles are found at near‐equal frequencies within the population 12, and probably evolved prior to divergence of humans and primates and the emergence of HLA class Ia genes 13, 14. Both HLA‐E*0101 and HLA‐E*0103 alleles have a relatively low expression on the cell surface 15. Although there is no structural difference of the peptide binding groove between these alleles, HLA‐E*0103 has a higher affinity for peptides than HLA‐E*0101, and is able to stabilize and up‐regulate at the cell surface much more efficiently 10, 16, 17. There is also variation in the peptide‐binding affinities of HLA‐E*0101 and HLA‐E*0103 in the absence of HLA class Ia and tapasin. In the absence of HLA class Ia peptides, for example due to viral down‐regulation, both alleles bind a diverse and mutually exclusive range of cellular‐derived peptides, which promote cell surface stability 18.

### Peptide binding and cell surface presentation

Unlike HLA class Ia molecules, the rigidity of the HLA‐E binding groove confers preferential avidity for a limited range of peptides. In a healthy cell setting, HLA‐E binds conserved leader peptides from HLA class Ia molecules 10, 19. The highly hydrophobic binding groove is ideally suited to bind a 9 amino acid ‘leader peptide’, typically VMAPRTL(L/V/I)L (VL9, Table 1) 20. Although VL9 is the optimal peptide for binding to HLA‐E, the affinity of the HLA‐E : peptide complex is defined by specific amino acid anchors; methionine (M) at position 2 and leucine (L) at position 9 21, with ancillary anchors at P3, P6 and P7 22. Higher‐affinity epitopes bind further into the HLA‐E binding groove, improving stability at the cell surface for longer 23. There are also several reported non‐MHC derived self‐peptides that contain a leader sequence able to up‐regulate HLA‐E on the cell surface (Table 2). HSP60 and adenosine triphosphate (ATP)‐binding cassette protein multi‐drug‐resistance protein (MRP)7 both contain putative leader peptides which demonstrate HLA‐E cell‐surface stabilization. However, the immunological function that these peptides exert from HLA‐E up‐regulation is currently unknown 18.

**Table 1:** Example human leucocyte antigen (HLA)‐E VL9 epitopes from HLA class Ia peptides 4, 19, 22, 25, 35, 84

Allele	Sequence
HLA‐A*0201	VMAPRTLVL
HLA‐A*01	VMAPRTLLL
HLA‐B*07	VMAPRTVLL
HLA‐B*27	VTAPRTVLL
HLA‐C*07	VMAPRALLL
HLA‐G*01	VMAPRTLFL

**Table 2:** Reported HLA‐E peptides found in pathogens and self‐peptides 18, 36, 61, 74, 100, 101

Pathogen	Gene product	HLA‐E leader peptide
HCMV	UL40	VMAPRTL(I/V/L)L
Hepatitis C virus	Core	YLLPRRGPRL
Epstein–Barr virus	BZLF1	SQAPLPCVL
HIV	P24	AISPRTLNA
*Mycobacterium tuberculosis*	Mtb14, P49, Mtb44	RMAATAQVL, RMPPLGHEL, RLPAKAPLL
*Salmonella typhimurium* serovar Typhi	GroEL	GMQFDRGYL

	Self‐peptides	
n.a.	HSP60	QMRPVSRVL
n.a.	ATP‐binding cassette protein MRP7	ALALVRMLI

HLA = human leucocyte antigen; HCMV = human cytomegalovirus; HSP = heat shock protein; ATP = adenosine triphosphate; MRP = multi‐drug‐resistance protein; n.a. = not available.

### HLA‐E orthologues

HLA‐E has defined orthologues within mammals, and is unusually conserved in function across the evolution of adaptive immune genes 24. In mice and rats, the corresponding HLA‐E orthologues are Qa‐1b and RT‐BM1, respectively 25, and Mamu‐E is the HLA‐E homologue in the rhesus macaque (*Macaca mulatta*) 24. Primate MHC is more polymorphic than human MHC, and Mamu‐E itself exhibits further polymorphism than human HLA‐E, with at least 33 functional alleles identified in rhesus macaque populations 24, 26. However, Mamu‐E is still the most conserved of all the class I MHC loci in macaques. HLA‐E and Mamu‐E share 88% amino acid identity, especially within the peptide binding region of the protein 27, and there is conservation in function between rhesus macaques, cynomolgus macaques (*M. fascicularis*) and humans 26, 27. Mamu‐E is also able to bind a wider range of peptides than HLA‐E, although it still preferentially binds the canonical VL9 peptide 5.

### HLA‐E function in a healthy cell – the fringe of innate and adaptive immunity

HLA class Ia nascent peptides are cleaved by signal peptidase, and are assembled and translocated in the endoplasmic reticulum (ER) of a cell via the peptide‐loading complex consisting of transporter associated with antigen processing (TAP), tapasin and calreticulin 28. HLA‐E epitopes from these peptides are cleaved by signal peptide peptidase, and bind to nascent HLA‐E within the ER, also via interaction with TAP and tapasin 25. On the cell surface, HLA‐E is stabilized by association with β2‐microglobulin 28, and predominantly interacts with CD94/NKG2 receptors on natural killer (NK) cells 15.

### HLA‐E and NK cells

Although classified as part of the innate immune system, NK cells span the traditional boundaries of innate and adaptive immunity through generation of memory‐like phenotypes and adaptation to infection 29, 30. They are defined through expression of the cell‐surface molecule CD56 31, and are vital for protection against viral pathogens, especially herpesviruses 19. NK cells target infected cells directly through the up‐regulation of inflammatory markers on the cell surface, or indirectly through their down‐regulation of MHC class Ia 32. HLA‐E, when stabilized with HLA class Ia‐derived peptides, exerts a regulatory function upon NK cells expressing the dimeric receptors CD94 and NKG2A or NKG2C 22, 33, 34. This interaction is dependent on the amino acid residues at positions 5 and 8 of the HLA‐E‐bound leader peptide 35. NKG2A is a C‐type lectin‐like receptor containing an inhibitory immunoreceptor tyrosine‐based inhibition motif (ITIM) sequence 36, which recruits Src homology 2 domain‐containing protein tyrosine phosphatase (SHP)‐1/2 tyrosine phosphatases 37 and prevents a release of cytotoxic granules containing interferon (IFN)‐γ and tumour necrosis factor (TNF)‐α 38. The HLA‐E : class Ia peptide complex can also bind the activating NK cell receptor CD94/NKG2C 39, albeit with an approximately sixfold lower affinity 36, 40. One exception is the HLA class Ib molecule HLA‐G, the VL9 peptide of which has phenylalanine at position 8, and interacts with a much higher affinity with CD94/NKG2C molecules 35. HLA‐G*01 is expressed during development of the placental trophoblast and has the ability to activate cytolytic CD94/NKG2C NK cells, potentially acting to maintain balance of tissue growth 35. CD94/NKG2 NK cell receptors have homologues in macaques and homoplasious molecules in mice 36, all of which interact with the corresponding orthologue of HLA‐E. Up‐regulation of HLA‐E by MHC class Ia peptides facilitates the passive monitoring of epitope presentation and TAP function within the cell; pathogen‐induced cessation of HLA class Ia synthesis prevents HLA‐E cell‐surface expression, and leads to destruction of the cell through ‘missing self’ activation of NKG2C^+^ NK cells 41.

## HLA‐E and infection

### Herpesvirus infection

Herpesviruses exhibit convergently evolved mechanisms that alter MHC presentation of viral antigens to the host adaptive immune system. Cytomegalovirus (CMV) is a highly species tropic β‐herpesvirus, and has evolved and diversified in tandem with its mammalian hosts 42. Human CMV (HCMV) is prevalent in 60–100% of a given population 43. HCMV manifests a lifelong latent infection, with subclinical presentation and immune control in immunocompetent individuals 1, 44. Infection is established within salivary gland epithelial cells 45, and disseminates throughout the host during latency 46.

**Figure 1:**
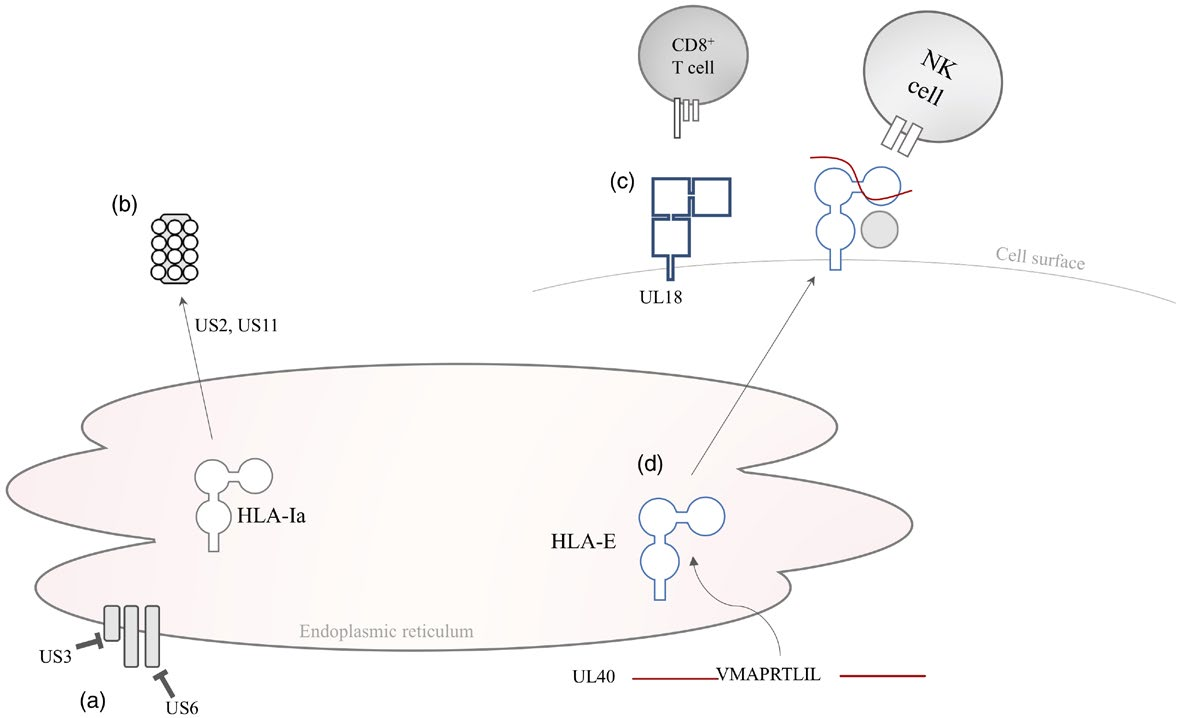
Manipulation of human leucocyte antigen (HLA) molecules by human cytomegalovirus (HCMV). (a) US3 and US6 prevent peptide binding to the HLA class Ia groove via interaction with tapasin and transporter associated with antigen processing (TAP), respectively. (b) US2 and US11 direct nascent HLA class Ia to the proteasome. (c) UL18 acts as a functional homologue of HLA‐E, and binds to the inhibitory leucocyte immunoglobulin‐like receptor 1 (LIR1) on T cells. (d) UL40 contains a VL9 leader peptide, which binds and stabilizes HLA‐E on the cell surface to interact with inhibitory CD94/NKG2A receptors on natural killer (NK) cells 1, 47, 48, 49, 50, 51, 52.

HCMV has evolved the ability to evade the host adaptive immune system through manipulation of HLA expression. The HCMV US2‐11 protein(s) down‐regulate HLA class Ia molecules on the cell surface 47, preventing presentation of viral epitopes to canonical CD8^+^ T cells (reviewed in Table 3). US2 and US11 induce translocation of HLA class Ia towards the proteasome 48, 49, US6 binds and changes TAP conformation to prohibit peptide binding to HLA class Ia 50 and US3 prevents peptide stabilization in the binding groove of HLA class Ia through direct binding to tapasin 51. Furthermore, UL18 is a homoplasious protein with similar function to HLA class Ia molecules. UL18 complexes with β2‐microglobulin on the cell surface and binds with high affinity to inhibitory leucocyte immunoglobulin‐like receptor 1 on T cells, down‐regulating their cytotoxic activity 52 (Fig. 1).

**Table 3:** The HCMV and RhCMV gene products involved in MHC manipulation 47, 48, 49, 50, 51

Gene product in HCMV	Gene product in RhCMV	Effect on MHC expression
	Down‐regulation of MHC class Ia on the cell surface	
US2, US11	Rh182, Rh189	Retrotranslocation of MHC class Ia from endoplasmic reticulum to cytoplasm, for degradation in the proteasome
US6	Rh185	Alters TAP conformation and peptide binding to MHC class Ia groove
US3	Rh184	Interacts with tapasin and prevents peptide binding to MHC class Ia groove
	Up‐regulation of MHC class Ib/prevention of cytotoxic responses	
UL18	Not present	HLA class Ia functional homologue that can bind inhibitory LIR1 T‐cell receptor
UL40	Rh67	Stabilizes and up‐regulates MHC‐E at the cell surface

HLA = human leucocyte antigen; HCMV = human cytomegalovirus; LIR1 = leucocyte immunoglobulin‐like receptor 1; MHC = major histocompatibility complex; RhCMV = rhesus cytomegalovirus; TAP = transporter associated with antigen processing.

Of most relevance to HLA‐E is UL40, a 221 amino acid glycoprotein containing a 37 amino acid signal sequence with an HLA‐E binding VL9 leader peptide that is identical to the leader peptide of HLA‐C*03 (Tables 1 and 2) 1, 19, 47. Although the function of the UL40 protein is unknown, the UL40 leader peptide binds to nascent HLA‐E in the endoplasmic reticulum in a TAP‐independent manner, working synergistically with the US6 family of HCMV gene products that inhibit TAP function 1, 53. Crucially, the UL40‐VL9 leader peptide is sufficient to up‐regulate HLA‐E expression on the cell surface 54, whereby it prevents NK cell cytolysis of infected cells through interaction with the inhibitory CD94/NKG2A molecule 1. This viral‐mediated up‐regulation of HLA‐E by UL40 can overcome the reduction of the VL9 leader peptide from down‐regulation and proteolysis of HLA class Ia peptides by HCMV. This, in turn, prevents CD94/NKG2A NK cell‐mediated killing of HCMV‐infected cells, despite the lack of HLA class Ia‐derived leader peptide. Loss of UL40 in the CMV genome leads to CD94^/^NKG2C NK cell‐mediated cytolysis of infected cells 54.

During the course of CMV infection, a subset of NK cells with low CD56 and high NKG2C expression (CD56^dim^NKG2C^bright^) are vastly expanded in approximately 50% individuals, and do not contract during latent infection. This NK subset has the potential to control the virus through increased cytotoxic activity 55. CD56^dim^NKG2C^bright^ NK cells may also express CD57, a marker of maturation and terminally differentiated NK cells 56, 57, which potentially induce a form of innate ‘memory’ towards persistent infections such as HCMV.

### HIV

Similarly, human immunodeficiency virus (HIV) causes a lifelong and latent infection, which is fatal if left untreated. HIV contains a putative HLA‐E binding leader peptide in the p24 gene product (AA9, Table 2). Although AA9 can stabilize HLA‐E on the cell surface, it is not sufficient to initiate interaction with CD94/NKG2 molecules on NK cells 58. Therefore, CD94/NKG2A^+^ NK cells are not inhibited by HLA‐E expression, and facilitate cytolytic killing of HIV‐infected cells 59. In HIV infection, there is an expansion of the NKG2C^bright^ NK compartment, although this has been correlated with HCMV co‐infection and not as a response to HIV 60.

### Mycobacterium tuberculosis

Tuberculosis afflicts roughly one‐third of the world’s population, and is caused by persistent latent infection by the bacterium *Mycobacterium tuberculosis *(*Mtb*) 61. The current licensed vaccine against *Mtb *infection is bacillus Calmette–Guérin (BCG), a live‐attenuated vaccine derived from *M. bovis* 62. NK cells form a substantial part of the innate immune response generated from this vaccine, and produce inflammatory cytokines in response to infection 63. Although understanding of NK cell function during *Mycobacteria *infection is limited, NK cells in the *Mtb *granuloma exert cytotoxic pressure through production of granulysin and perforin 64, as well as restriction of bacterial growth through direct contact with infected cells via cytotoxic NKG2D^+^ NK cells 32. HLA‐E : *Mtb *peptide complexes are not recognized by CD94/NKG2 molecules, so do not control activation or inhibition of NK cells through these receptors 65.

### NK cells and trained immunity

NK cells (and other innate lymphoid cells such as macrophages and monocytes) display ‘trained immunity’ in response to BCG vaccination 66. Trained immunity induces a lasting anti‐pathogen response to secondary, unrelated antigen exposure 62, which in NK cells correlates with increased proinflammatory cytokine production against new pathogens 67, 68. In infants, BCG vaccination correlates with increased weight gain, reduced mortality and reduced infection ability of other *Mycobacteria *species 66. In immunodeficient SCID mice lacking T and B cells, similar protection is observed after BCG vaccination towards *Schistosoma *and *Candida *infection 66, 69. The proinflammatory effect of BCG is also commonly used to treat urothelial cell carcinoma 70. Trained immunity differs from innate memory, as the heightened response is not specific to the original pathogen, although is stronger compared to antigen‐naive innate cells 62 and occurs as a result of histone methylation at the H3K4me1 locus of innate immune cells, inducing a lasting enhanced level of NK cell activation and cytokine production 56, 63, 71.

## HLA‐E and T cell restriction

HLA‐E‐mediated presentation of pathogen‐derived peptides to T cells has been observed during infection with CMV, *Mtb* and *Salmonella enterica* 72, 73, 74, and recently in CMV‐vectored vaccines against simian immunodeficiency virus (SIV) 5.

### Mycobacterium tuberculosis

In humans and mice, CD4**^+^** and CD8^+^ T cells are vital for control of *Mtb* infection 75. Unusually, for any known pathogen, there is a large population of CD8^+^ T cells restricted by HLA‐E induced by infection 76, possibly enhanced by the up‐regulation of HLA‐E on the surface of *Mtb‐*infected phagosomes 65, 70. Multiple peptides within the *Mtb *genome can be presented (including peptides from p49 and Mtb44 proteins, Table 2) 61, 72, 74. Their varying amino acid length implies that HLA‐E has higher peptide binding plasticity than solely the VL9 peptide 75. These non‐canonical T cells contribute to the majority of T cells present during active *Mtb* infection 77, and overshadow T cells restricted by canonical HLA class Ia epitope presentation 78, 79. *Mtb* antigens presented by HLA‐E to CD8^+^ T cells can induce either a cytotoxic or regulatory phenotype, consequently inhibiting *Mtb* pathogenesis and growth in infected macrophages 7, 78. HLA‐E : *Mtb*‐restricted T cells from active TB infection express a type 2 cytokine profile with increased interleukin (IL)‐4 and IL‐10 production, and assist B cells with antibody and cytokine production to inhibit *Mtb *growth 65, 76, 77.

### Cytomegalovirus

Classically restricted HCMV‐targeting CD8^+^ T cells are critical in the control of HCMV infection, and constitute up to 10% of the circulating T cell population during active infection 46, 48, 80, typically directed towards epitopes in the pp65 and IE peptides 81. Infection with HCMV also establishes a ‘memory inflation’ population of CD8^+^ effector memory T cells (T_EM_), which are defined by their large expansion after infection, terminally differentiated phenotype (CD57^+^) and their expression of CX3CR1 81, 82, 83. In CMV‐seropositive individuals, a proportion of CD8^+^ T cells are HLA‐E‐restricted, long‐lasting and express a T_EM_ phenotype 83, although interact with the T cell receptor (TCR) with much lower affinity than HLA class Ia‐bound peptides. These T cells recognize the UL40‐VL9 epitope presented by HLA‐E, and may arise from permanent exposure to HCMV epitopes, which perpetuate a stronger response comparable to canonically restricted CD8^+^ T cells 83, 84.

## Utilizing unusual immune phenotypes for vaccine development

The role of HLA‐E in NK cell activation has been well studied; however, the unforeseen property of HLA‐E to restrict non‐canonical T cells has revealed interesting potential for developments in vaccine design and efficacy.

### CMV‐vectored vaccines for SIV

Cytomegalovirus, when used as a vaccine vector in rhesus macaques, induces the restriction of non‐classical T cells by Mamu‐E. This has been demonstrated in a rhesus macaque CMV‐vectored vaccine against SIV, the primate homologue of HIV 85. SIV, like HIV, is a lentivirus that permanently infects the host through integration into the genome**. **SIV has been used as a close model for HIV, and causes similar pathogenesis including loss of CD4^+^ T cells, AIDS‐like illness and eventually death in infected macaques 86.

CMV as a virus, and as a vaccine vector, can generate swift T_EM_ cell responses at the site of exposure 87, 88. In contrast, many non‐viral‐vectored vaccines activate a slower‐acting T central memory (T_CM_) response in the secondary lymphoid organs 89. In these studies, rhesus macaque cytomegalovirus strain 68‐1 (RhCMV 68‐1) was used as a vaccine vector, in accordance with the highly species tropic nature of cytomegaloviruses. The SIV genes gag+rev/nef/ tat+env+pol were expressed in this RhCMV vector 87, 90. In all vaccinated macaques, this vaccine generated robust, long‐lasting CD4^+^ and CD8^+^ T_EM_ responses to all SIV peptides, contrasting with predominantly T_CM_ responses from adenovirus‐vectored vaccines containing the same peptides. Fifty‐five per cent of RhCMV : SIV vaccinated macaques generated completely protective immune responses when challenged 59 weeks later with intrarectal infection of highly pathogenic SIVmac239 88. These atypical CD8^+^ T cell responses were able to recognize broad SIV epitopes, alongside occasional ‘supertopes’ (epitopes recognized by all macaques) within the vaccine, regardless of the MHC haplotype of the macaques 90. Furthermore, classically defined SIV epitopes were not recognized by the T cells of any macaque. Protection was also conferred irrespective of the route of SIV infection, and could be transferred to SIV‐seronegative macaques after adoptive transfer of haematolymphoid cells prior to SIVmac239 challenge 86.

### The RhCMV : SIV vaccine restricts non‐canonical CD8^+^ T cells

In macaques vaccinated with the RhCMV : SIV vaccine, MHC class II‐restricted T cells made up 65% of this non‐canonical response, and the remaining 35% were restricted by Mamu‐E. No canonically restricted CD8^+^ T cells were produced in response to this vaccine 5, 90. The Mamu‐E‐restricted SIVgag‐specific response was so broad that an average of 20 SIVgag epitopes were recognized per vaccinated macaque, in contrast to ~13 SIVgag epitopes from canonical CD8^+^ T cell restriction 90. This broad response in the non‐canonical CD8^+^ T cell compartment has not been previously observed in any preclinical vaccine trial. It may be due to the deletion of Rh157.4 and Rh157.5, which encode an orthologue of the HCMV pentameric glycoprotein receptor complex encoded by UL128 and UL130, which enables non‐fibroblast tropism 5, 90. Similarly to HCMV, normal RhCMV infection only activates canonically restricted T cells 91, and due to the differences between the vaccine and wild‐type RhCMV there is no pre‐existing immunity against this RhCMV‐vectored vaccine in macaques 92.

### RhCMV contains an MHC‐E‐binding VL9 sequence

Through this RhCMV‐vectored SIV vaccine, Mamu‐E exhibits the ability to overcome the classical paradigm of MHC class Ia T cell restriction, and in doing so generates an enormous breadth of non‐canonical T cell responses. This, in theory, can offer equal protection to all vaccinated individuals, regardless of MHC genotype, and suggests that Mamu‐E can bind a much broader range of peptides beyond the canonical VL9 leader peptide 5. The VL9 peptide in this vaccine is provided by the RhCMV gene Rh67, which is loaded into Mamu‐E in a TAP‐independent manner. Rh67 initiates Mamu‐E up‐regulation at the cell surface in a convergent fashion to HCMV‐UL40. It is hypothesized that the Rh67‐VL9 peptide can bind and stabilize Mamu‐E deep in the rigid, hydrophobic binding groove, acting as a chaperone and allowing a broader range of SIVgag epitopes to bind higher up in the binding groove and interact with Mamu‐E‐restricted T cells. Although cytomegaloviruses have high species tropism, Rh67 can stabilize and up‐regulate HLA‐E in human cells, suggesting that the function of the ancestral MHC‐E gene has remained conserved 5. This suggests that in the RhCMV : SIV vaccine, Rh67 is facilitating the broad peptide presentation by stabilization of Mamu‐E on the cell surface, allowing presentation of antigen peptides to non‐classical CD8^+^ T cells. Furthermore, several MHC‐altering genes in RhCMV and HCMV share surprising functional similarity through preventing cell‐surface presentation of MHC class Ia molecules 48 (Table 3). Overall, the convergent evolution of RhCMV‐Rh67 and HCMV‐UL40, and other genes involved in MHC down‐regulation, suggests that the mechanism of HLA‐E stabilization is conserved between cytomegalovirus species, and therefore a HCMV‐vectored vaccine in humans may function in the same way.

### CMV‐vectored vaccines for tuberculosis

Recently, Hansen *et al.* 93 published a preclinical trial testing three RhCMV strain 68‐1‐vectored vaccines expressing six or nine proteins from *Mtb*. All vaccines induced IFN‐γ‐ and TNF‐producing T_EM_ cells that are vital in protection against *Mtb* infection, and induced complete or partial protection in > 40% vaccinated macaques. One vaccine, using the original RhCMV68‐1 vector from the SIV vaccine challenge, elicited unconventionally restricted MHC class II‐ and MHC‐E‐restricted T cells. However, two new ‘68‐1.2 RhCMV’‐vectored vaccines only exhibited a canonical T cell response in vaccinated macaques. All three vaccines, however, obtained the same levels of efficacy during challenge trials, suggesting that, contrary to earlier reports 61, 65, Mamu‐E‐restricted T cell responses are not vital for control of *Mtb *infection 93. Natural *Mtb *infection induces both cytotoxic and regulatory‐like HLA‐E‐restricted T cells in humans. HLA‐E‐restricted regulatory T cells are likely to be beneficial in containing the *Mycobacterium *and preventing dissemination 61, 76. However, HLA‐E‐mediated peptide presentation may not be as beneficial to *Mtb *vaccine function if the induction of regulatory CD8^+^ T cells reduces the progression of disease, but does not establish a cytolytic environment targeted to *Mtb *infection. It will also be interesting to see if, in future studies, this *Mtb *vaccine can generate similar trained immunity in innate and NK cells, as is seen in the current BCG vaccine.

### CMV vaccines in human clinical trials

Phase I clinical trials of CMV vaccines in humans were not able to elicit the same broad epitope response as RhCMV‐vectored vaccines. The vaccine, created from chimeric Towne and Toledo fibroblast‐adapted strains, was aimed at inducing immunity against HCMV infection, rather than for use as a viral vector. This vaccine did not contain the pentameric glycoprotein complex, facilitating non‐fibroblast trophism in a similar fashion to the RhCMV vector. However, vaccination did not elicit any non‐canonically restricted T cells in the human participants 94, 95, 96. There is also reasonable concern regarding the use of CMV as a vaccine in CMV‐seropositive individuals, thus creating a ‘superinfection’ serostatus, and possibly negating the immunogenic effect of the vaccine. Although this proved unproblematic in macaques, which have a RhCMV seropositivity of > 90% in captive populations 97, it is still uncertain what effect this will have in human clinical trials, given the ability of HCMV to reduce vaccine efficacy 98.

## Future directions for utilizing pathogens to enhance vaccine efficacy

The dual functionality of HLA‐E, through its ability to inhibit NK cells and activate non‐classical CD8^+^ T cells, has made it an intriguing target of research for both understanding the immunology behind pathogen infection and improving vaccine design. Although it is attractive to think that the results obtained by Hansen *et al.* 5, 87, 88 in the SIV field could lead to the creation of a non‐conventional T cell stimulating vaccine in humans, there are still fundamental questions that must be answered to assess the potential for and safety of developing a vaccine that can induce HLA‐E restricted T‐cells. It is also important to acknowledge the existence of self‐peptides, including HSP60, that contain potential HLA‐E leader peptides 99. These peptides are able to up‐regulate HLA‐E, although their immunological function is not well understood, and therefore may have an impact on vaccine efficacy.

However, as HLA‐E is emerging as an important aspect of the host response to several pathogens, understanding how it can restrict T cells via non‐conventional mechanisms will continue to be an important avenue of vaccine development, and also improve fundamental understanding of how HLA‐E borders innate and adaptive immunity. Furthermore, the important contribution of NK cells towards vaccine efficacy has still to be fully elucidated.

In conclusion, trained immunity and the restriction of non‐canonical CD8^+^ T cells by CMV‐vectored vaccines are just two examples of unexpected effects caused by vaccination, which are impacting future vaccine design. If viral‐vectored vaccines can be developed to induce HLA‐E‐restricted T cells in human patients, it may pave the way for the development of vaccines with broad, fast‐acting and best‐placed immunogenicity against many pathogens.

## Disclosure

The authors declare no competing interests.

## Author contributions

H. R. S. and T. L. conceptualized and wrote the review; G. B. and S. B. contributed to the review structure, contents and proofreading.
